# Significant changes in brain hemodynamic status in the presence of β‐amyloid deposition identified by Quantitative Gradient Recalled Echo (qGRE) MRI

**DOI:** 10.1002/alz.095350

**Published:** 2025-01-09

**Authors:** Satya V.V.N. Kothapalli, Tammie L.S. Benzinger, Andrew J. Aschenbrenner, Cihat Eldeniz, John C. Morris, Dmitriy A. Yablonskiy

**Affiliations:** ^1^ Washington University School of Medicine in St. Louis, St. Louis, MO USA; ^2^ Knight Alzheimer Disease Research Center, St. Louis, MO USA; ^3^ Hope Center for Neurological Disorders, Washington University in St. Louis, St. Louis, MO USA

## Abstract

**Background:**

There is a growing interest in investigating cerebrovascular dysfunction related to early Alzheimer disease (AD) pathology. The objective of this study is to evaluate changes in brain hemodynamic properties in relation to excess of β‐amyloid (Aβ) deposition using the quantitative Gradient Recalled Echo (qGRE) MRI technique. Additionally, we aim to assess brain Aβ status by combining output metrics of qGRE and quantitative susceptibility maps (QSM).

**Method:**

43 amyloid positive (Aβ^+^, mean age 74.28), and 62 amyloid negative (Aβ^−^, mean age 72.02) participants were recruited by Knight ADRC. qGRE and QSM images were obtained from the same 3D multi‐gradient echo sequence using 3T Siemens MRI scanners. R2' metric of qGRE signal defines regional concentration of deoxyhemoglobin (Ulrich&Yablonskiy, MRM‐2016). Combining qGRE and QSM data allows separate evaluation of deoxyhemoglobin and tissue contributions to QSM metric (Yablonskiy, et al, Neuroimage‐2021). The principal component analysis (PCA) for the whole grey matter and lobe‐wise analysis (medial temporal (MTL), temporal, parietal, occipital, and frontal lobes) were used to present data.

**Result:**

Statistical analysis for the whole grey matter shows increased deoxyhemoglobin concentration (p = 0.0003) in Aβ^+^ as compared to Aβ^−^ groups. This increased deoxyhemoglobin was detected in all lobes: MTL (p = 0.001), temporal (p = 6.5E‐5), parietal (p = 0.006), occipital (p = 0.02), and frontal (p = 0.008). Data also showed brain‐wide decreased in tissue susceptibility (p = 0.01) in Aβ^+^ as compared to Aβ^−^ groups, though lobe‐wise difference reached statistical significance only in temporal (p = 0.01) and frontal (p = 0.04) lobes.

**Conclusion:**

Our results indicate that participants with excessive amyloid deposition exhibit an increased concentration of deoxygenated blood. Importantly, the odds ratio of being amyloid positive in the presence of higher concentration of deoxyhemoglobin is increased by a factor about five. Detail analysis of qGRE data shows that increased concentration of deoxyhemoglobin is mostly caused by increased of the deoxyhemoglobin‐carrying blood vessel network (venous blood and pre‐venous part of the capillary bed). In MTL and temporal lobes qGRE also detected significant increases in oxygen extraction fraction (OEF). Additionally, discovered reduced tissue magnetic susceptibility in Aβ^+^ participants is indicative of increased Aβ deposition, thus providing new MRI‐based tool for identifying brain tissue Aβ status.

